# The Effect of Probiotics Use on Salivary Cariogenic Bacteria in Orthodontic Patients with Various Caries Risk Status

**DOI:** 10.3390/nu14153196

**Published:** 2022-08-04

**Authors:** Liang-Ru Chen, Chia-Li Lai, Jun-Peng Chen, Chia-Tze Kao

**Affiliations:** 1 Pediatric Dentistry and Orthodontics, Department of Stomatology, Taichung Veterans General Hospital, Taichung 40705, Taiwan; 2 School of Dentistry, National Yang Ming Chiao Tung University, Taipei 112, Taiwan; 3 Orthodontic Department, Chung Shan Hospital, School of Dentistry, College of Oral Medicine, Chung Shan Medical University, Taichung 40201, Taiwan; 4 Department of Medical Research, Taichung Veterans General Hospital, Taichung 420, Taiwan

**Keywords:** probiotics, orthodontics treatment, caries risk assessment

## Abstract

Background: The purpose of this study was to evaluate the change in intraoral cariogenic bacteria density after probiotic use in patients with orthodontic treatment, and to compare the impact of probiotics in patients with various caries risk status. Methods: Patients that planned to receive orthodontic treatment were recruited according to this study’s inclusion/exclusion criteria. A probiotic prescription (Lactobacteria 3 mg, Glycobacteria 2 mg) was started one month after the initial orthodontic treatment. Saliva sampling and cultures using a CRT kit (caries risk test) were performed at three time points (T0, T1, T2). Mutans streptococci (MS) and Lactobacilli (LB) density were evaluated and scored using the interpretation chart in the CRT kit to evaluate the change in bacteria density at three time points, to define the high and low caries risk prior to orthodontic treatment, and to evaluate if there were differences in probiotics between the high and low caries risk groups. Results: Thirty-three orthodontic patients were enrolled, twenty-two classified as high caries risk and eleven as low caries risk. After undergoing treatment for one month, the densities of MS and LB increased significantly (*p* = 0.011, *p* = 0.001); probiotics for one month decreased the density of MS and LB, but the differences were statistically nonsignificant (*p* = 0.109, *p* = 0.109). Patients classified as low risk of caries demonstrated an increase in MS and LB density one month after orthodontic treatment (*p* = 0.024, *p* = 0.001), probiotic use did not result in a significant reduction in bacteria density (*p* = 1000, *p* = 0.933). In patients with high caries risk, there were no statistically significant changes in MS count between the three time points (*p* = 0.127); a significant change in LB density occurred at T0–T1 (*p* = 0.011) only. Conclusions: Supplemental use of probiotic oral tablets during orthodontic treatment aimed at reducing cariogenic bacteria count in saliva did not achieve significant differences, regardless of patients’ risk status for caries.

## 1. Introduction

The benefits of probiotics for human health have been discussed for decades. Probiotics are non-pathogenic organisms which, when administered in an adequate amount, interact directly or indirectly with pathogens in the intestine, resulting in a more natural method for restoring the microbiota and conferring a health benefit to the host [1]. Possible mechanisms contributing to the regulation of intestinal pathogenic microorganisms through the use of probiotics include coaggregation, biosurfactant production, bacteriocin and H_2_O_2_ production, signaling effects, competitive exclusion, immunomodulation, and modulation of tight junctions [2]. The effects of probiotics on oral diseases have also been addressed [3,4], since dental caries and periodontal diseases, the two most common oral diseases, have been shown to be related to a bacterial ecologic shift from normal flora to pathogen-dominant environments in oral cavities. Obvious examples are mutans streptococci (MS), a key microorganism in the initiation of caries, and lactobacilli (LB), which contributes to the further development of caries [5]. Probiotics have long been used in various kinds of products (e.g., tablets, powder, tooth, milk, and salt). The mechanism by which probiotics confer a beneficial effect on intraoral microorganisms is thought to be comparable to the mechanisms at play in the intestines, with interactions of microorganisms at both systemic and local levels [6]. The findings of a review of the literature on probiotic bacteria as a potential anti-caries measure conducted by Twetman and Keller did not exclude the possibility that probiotic bacteria could interfere with oral biofilm [7]. In a series of systematic reviews on probiotics use in caries prevention, salivary mutans streptococci levels were found to be reduced by short-term probiotic supplements [8,9]. In a systematic review and meta-analysis [10] dairy probiotics were reported to be effective in reducing streptococcus mutans, increasing salivary pH and promoting a higher plaque index. 

During the traditional orthodontic treatment process, food debris readily accumulates on the surface of the tooth due to the presence of brackets and wires [11]. In spite of the provision of routine oral hygiene care instruction at every visit, an increased incidence of white spot lesions, dental caries, or gingival inflammation is frequently observed. It was interesting to know whether probiotics can help improve oral health during orthodontic treatment. In the systemic review by Hadi–Hamou et al., they incorporated the conclusion that supplementation of orthodontic patients did not affect the development of inflammation in the gingiva and decalcification of the enamel [12]. Gizani et al. studied the effect of probiotic bacteria on the development of white spot lesions in orthodontic patients [13]. They found that there were no differences in the incidence of white spot lesions between groups at debonding, and the levels of salivary LB levels were significantly reduced in both groups at the time of debonding compared with baseline, while no alterations in MS counts were found. A study by Pinto et al. compared the use of yogurt with or without Bifidobacterium animalis subspecies in orthodontic patients [14]. Their results indicated that use of yogurt containing B. animalis subsp. lactis for 2 weeks was insufficient to reduce counts of MS and LB in the saliva or dental plaque of patients with fixed orthodontic appliances. A study by Alp evaluated the effect of probiotic kefir on streptococcus mutans and lactobacillus levels in orthodontic patients [15]. They concluded that systemic consumption or local application of probiotics during fixed orthodontic treatment resulted in lower levels of MS and LB in the saliva. However, studies on the effects of probiotic use in orthodontic patients are still limited, and the findings may be affected by the strains of probiotics used, the duration of probiotic use, and the different outcome variables used. The caries incidence of the orthodontic patients may also be related to individual caries risk status. Caries risk assessment in orthodontic patients was conducted in a study by Enerback et al. [16]. Their study focused on evaluating the reliability of various methods of caries risk assessment in orthodontic patients. Mulla’s study used a decayed filled surfaces index (DFS) and cariogram computer system to access a patient’s caries profile [17]. Their study concluded that patients with high caries risk before orthodontic treatment had a higher risk of developing caries. These patients had a significantly higher number of MS and LB in the saliva and had fewer chances of avoiding new cavities. Therefore, studies of probiotic use in orthodontic patients without considering individual caries risk status may lead to bias in the results. However, these kinds of studies were still rare. The purpose of this study was to evaluate the effect of the use of probiotics on intraoral cariogenic bacteria levels in orthodontic patients and to see if the impact was different in patients with various caries risk statuses.

## 2. Materials and Methods

### 2.1. Patient Selection

The patients were recruited from the Department of Pediatric Dentistry and Orthodontics of Taichung Veterans General Hospital. Patients who planned to receive comprehensive traditional orthodontic treatment and met the following criteria were included: (1) no history of previous partial or comprehensive orthodontic treatment; (2) no existence of untreated dental caries; (3) age between twelve and thirty years old. The exclusion criteria were: (1) patients with congenital or systemic disorders; (2) patients with immunocompromised conditions; (3) history of periodontitis; (4) women who were pregnant or lactating; (5) history of antibiotics or other medications prior to or during orthodontic treatment; (6) poor compliance with the use of probiotics (less than five days per week). Informed consent was obtained from all subjects involved in the study.

### 2.2. Power Analysis and Sample Size Estimation

The power analysis and sample size needed has been added in the method section. Power analysis was performed by G*Power Software. A total sample size of 28 participants would give 81.1% power for repeated measures analysis of variance with two groups and three time points to detect significant differences with a 0.25 effect size at the α = 0.05 significance level.

### 2.3. Study Design

#### 2.3.1. Bracket and Band Selection

The orthodontic brackets used in this study were the Mini-Wick system designed by G.R. Wick Alexander (Ormco corp. Orange, CA, USA). The characteristics of its design were the twin bracket on the upper anterior teeth and the single bracket with rotation wing in the lower anterior and the posterior teeth. There was usually one band in each quadrant of the dentition in every patient. O-ring or ligature wire was used for fixation of the orthodontic wire to the bracket.

#### 2.3.2. Saliva Sample Collection

Patients who were included served as their own controls. There were three periods of saliva sampling: T0 denotes the time before orthodontic treatment. At T0, a saliva sample was collected, comprehensive orthodontic banding and bonding was then applied, and oral hygiene care instruction was given. T1 denotes one month after starting comprehensive orthodontic treatment. A saliva sample was collected using a kit, according to the manufacturer’s instructions, and use of probiotics was started. A prescription method and checklist were given. T2 denotes two months after starting the orthodontic treatment and one month of probiotic use. A saliva sample was collected and the patient’s compliance with probiotics was checked. 

The study protocol was approved by the Human Investigation Committee’s Institutional Review Board (IRB CF19055B) at Taichung Veterans General Hospital, Taichung, Taiwan.

#### 2.3.3. Use of Probiotics

The brand of probiotics used in our study was Biofermin (Viomet, Veterans Pharmaceutical CO., Taoyuan, Taiwan), whose main components are Lactobacteria 3 mg and Glycobacteria 2 mg/Table Prescription and dosage followed the packet insert. The patients were instructed to chew the tablets thoroughly before swallowing and to take three tablets of probiotics each time, three times a day, starting at T1 (one month after starting comprehensive orthodontic treatment and saliva sampling). Patients’ compliance was evaluated using a record sheet and by counting the remaining tablets in the bottle, which was taken at the third appointment (T3). During the observation period, the patients were instructed not to use antibiotics or any other types of probiotics.

#### 2.3.4. Saliva Sampling Method

Patients were instructed not to eat, drink, or brush their teeth one hour before sampling. The chewing of paraffin wax for two minutes was used to stimulate saliva secretion. The sample was collected and transferred to the two-sided agar surface of a CRT^®^ bacteria kit (Caries Risk Test, Ivoclar, Vivadent, Schaan, Liechtenstein) using a pipette. The light green agar was used for incubation of Lactobacilli spp. (LB) in saliva; the deep blue agar was used for the incubation of mutans streptococci (MS) in saliva.

#### 2.3.5. Incubation Method

The test vial was placed upright in the incubator and incubated at 37 °C/99 °F for 48 h.

#### 2.3.6. Bacteria Colony Formation Density Scoring Method

**Figure 1:**
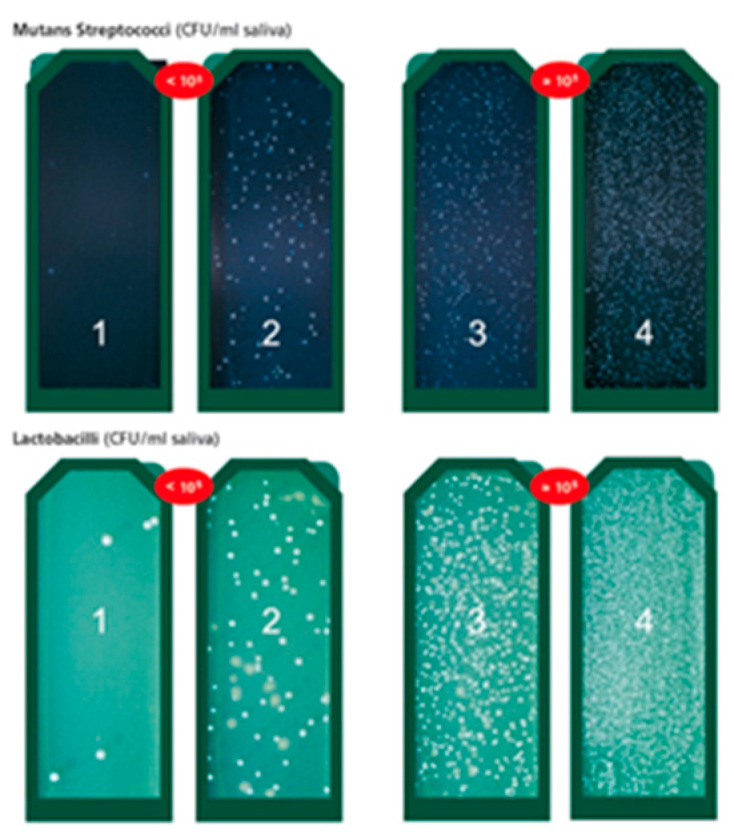
Interpretation chart of the bacteria density of the CRT^®^ kit: light green agar represented LB density; dark blue agar represented the MS density. Density less than 10^5^ CFU indicated low caries risk; density higher than 10^5^ CFU indicated a higher caries risk for both for MS and LB. The bacteria density was recoded as 1, 2, 3, or 4 for data analysis.

After removal of the vial from the incubator, the colony formation density of mutans streptococci (MS) and lactobacilli (LB) was interpreted using the evaluation chart provided by the CRT^®^ bacteria kit. Density of 10^5^ CFU or more of lactobacilli and mutans streptococci per ml saliva indicated a high caries risk. The evaluation chart was recoded from 1 to 4 for data analysis (Figure 1). To further analyze the characteristics of patients with high versus low caries risk in patients receiving orthodontic treatment and probiotic supplements, low caries risk patients were defined as having a MS colony formation density score (CRT score) equal to or less than 2 and a LB CRT score equal to or less than 2. High caries risk patients were defined as having a MS CRT score equal to or greater than 3 or a LB CRT score equal to or greater than 3.

### 2.4. Statistical Analysis

The changes of two cariogenic bacterial species colony formation density scores (CRT score) were analyzed using the Friedman test; multiple comparisons of change among the three time periods were analyzed using post-hoc analysis (Dunn-Bonferroni). 

Differences in gender, age, and bacteria count at different time points in the high and low caries risk groups were compared using the Mann–Whitney test. For further analysis of bacterial colony density change among the three time periods in the respective two caries risk groups, the Kruskal–Wallis test was used.

## 3. Results

This study enrolled 33 orthodontic patients (18 male and 15 female), with an average age of 15.8 years (range, 144 to 274 months old). The average age of male participants is 15.7 years (range, 150–245 months old) and the average age of female participants is 15.4 years (range, 144–274 months old). Changes of bacterial count scores over time and multiple comparison of scores among the three time points are illustrated in Table 1.

There were significant differences in CRT score changes (CRT^®^ bacteria kit evaluation chart with scoring in Figure 1) between the three time points for both Streptococcus mutans and Lactobacilli species (*p* = 0.001, *p* < 0.001) (Table 1). The bacteria count of both cariogenic bacteria significantly increased one month after bracket bonding and before probiotic use. The CRT scores slightly decreased after one month’s use of probiotics. However, the multiple comparison test indicated that the major changes of the CRT scores in both species occurred between time point T0 and T1 (*p* = 0.011, *p* = 001).

The distribution of the CRT scores in the low and high caries risk groups are illustrated in Table 2. Eleven subjects were classified as having a low caries risk and twenty-two subjects were classified as having a high caries risk. There were no significant differences in the gender ratio (*p* = 0.711) or age (*p* = 0.576) between the two groups. At time point T0, the low caries risk group had significantly lower scores in both cariogenic bacteria species compared with the high caries risk group (*p* < 0.001, *p* < 0.001). However, the differences in bacteria scores between the two groups became non-significant at time points T1 and T2. 

**Figure 2:**
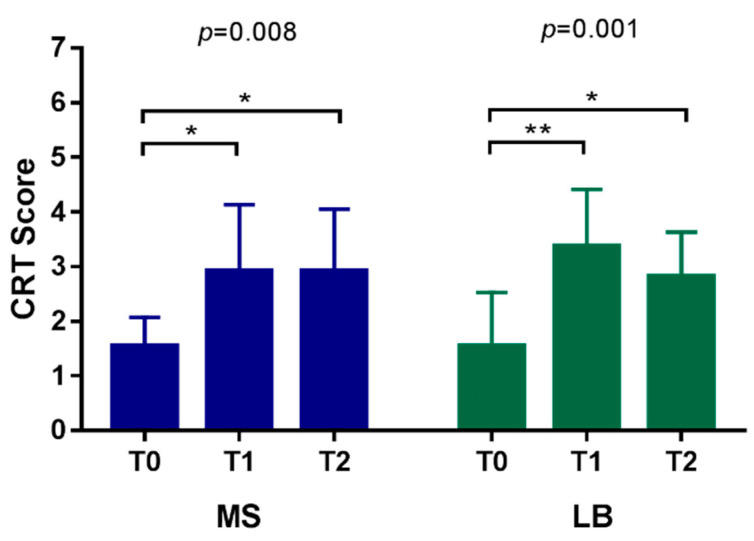
CRT score of the low caries risk group among three time point. Multiple comparison test. * *p* < 0.05, ** *p* < 0.01.Table 4 and Figure 3 present the changes in the cariogenic bacterial count among the three time points in the high caries risk group. There were no statistically significant changes in the MS count among the three time points; for LB count, there were significant changes among the three time points (*p* = 0.014). Multiple comparisons revealed that significant changes occurred at T0–T1 (*p* = 0.011).

Table 3 and Figure 2 present the changes in cariogenic bacterial counts among the three time points in the low caries risk group. There were significant changes in streptococcus mutans count between the three time points (*p* = 0.008). Multiple comparisons revealed that changes occurred at T0–T1 and T0–T2 (*p* = 0.024, *p* = 0.002, respectively); for the LB count, there were significant changes among the three time points (*p* = 0.001). Multiple comparisons revealed that significant changes also occurred at T0–T1 and T0–T2 (*p* = 0.001, *p* = 0.021, respectively). Probiotics use had no significant effect on low caries risk patients, and no significant differences were found between T1 and T2 for both species of bacteria. 

## 4. Discussion

### 4.1. Changes in Cariogenic Bacterial Count Scores in Orthodontic Patients over Time

Some previous systematic reviews and meta-analyses in the literature have concluded that regular intake of probiotic products may decrease MS counts of plaque and saliva in the short-term [8,9]. However, in patients receiving orthodontic treatment, the intraoral environment and bacterial ecology might be different, and thus, the same trends may not be observed. Dental plaque accumulation may occur more readily if no additional effort is made. In the first part of this study, patients served as their own control. The change in CRT score between time points T0 and T1 represented the change in intraoral cariogenic bacterial density after orthodontic treatment, and the change between time points T1 and T2 represented the effect of the intervention of probiotics. Table 1 shows there were significant differences in CRT score changes among the three time points in both streptococcus mutans and lactobacilli species, and the changes occurred mainly between time points T0 and T1 (T0: no intraoral orthodontic brackets/bands/wires; T1: four weeks after placing brackets/bands/wires and before probiotics use). This meant that placing brackets in orthodontic patients changed the intraoral bacterial ecology, and the bacteria density increased for both cariogenic bacteria. One month of probiotic use (T1 to T2) decreased the bacteria density for both cariogenic bacteria, however, the differences were not statistically significant. This result is consistent with the findings of Ginazi et al., whose study used Lactobacillus reuteri as the intervention probiotic and white spot lesions as the outcome variable [13]. They concluded that daily intake of probiotic lozenges did not appear to affect the development of the white spot lesions during orthodontic treatment with a fixed appliance. Their study also indicated no significant changes in the levels of salivary MS after probiotic intervention. The results of our study were not consistent with Alp and Baka’s findings, which showed regular use of probiotics during fixed orthodontic treatment reduced MS and LB levels in the saliva [15]. Our study differed from the study by Alp and Baka in four key aspects: (1) The saliva sample was collected prior to placement of orthodontic appliances into the oral cavity (T0) in our study, and this served as the baseline data of the patients who received orthodontic treatment, i.e., they served as their own control, while in Alp and Baka’s study, the beginning of their study (T0) was set three months after full mouth bandings and bondings; (2) Orthodontic materials such as O-ring, figure eight ligature wires, and coil spring were not used in Alp and Baka’s study out of a concern for possible adverse effects on oral hygiene; our study used these orthodontic materials in order to mimic the real intraoral environments of orthodontic treatment; (3) the final timing of saliva sampling in Alp and Baka’s study was 6 weeks after the use of probiotics, while our study collected the final saliva sample four weeks after probiotics use; (4) different species of probiotics were used. In short, the results of cariogenic bacterial count change following orthodontic treatment may vary a lot due to the use of different study designs.

### 4.2. The Effect of the Use of Probiotics in Orthodontic Patients with Low Caries Risk

In the second part of the study, 11 patients were classified as having a low caries risk and 22 patients had a high caries risk at the beginning of the study (Table 2). The low caries risk group had significantly lower scores for both cariogenic bacteria species compared with the high caries risk at time point T0, which was consistent with the original definition of low/high caries risk. However, at time point T1 (after orthodontic treatment for one month and before probiotics supplements) and at T2 (one month after probiotics supplements), the differences in bacteria scores between the two groups became nonsignificant. This implies that there were significant ecologic changes in cariogenic bacteria even in low caries risk orthodontic patients. The intervention of probiotics use did not provide any benefits to the low caries risk group. The characteristics of the low caries risk group after using probiotics were further analyzed, and the results are shown in Table 3. It was evident that there were significant ecologic changes in the cariogenic bacteria in the low caries risk group of the orthodontically treated group. Both the Streptococcus mutans and Lactobacilli species counts changed significantly among the three time points, and the changes occurred mainly in the T0–T1 and T0–T2 intervals. Clinically, this means that, even if a patient was classified as having a low caries risk prior to orthodontic treatment, the placement of brackets/bands/wires in the oral cavity might still have increased the cariogenic bacteria counts (T0 to T1). This interesting phenomenon warrants further study. After using probiotics for one month (T1–T2), the bacteria count did not reveal significant changes, indicating the probiotics did not have a significant impact on the cariogenic bacteria ecology in the low caries risk group.

### 4.3. The Effect of the Use of Probiotics in Orthodontic Patients with High Caries Risk

The characteristics of the high caries risk group after using probiotics were further analyzed, and the findings are shown in Table 4. The data revealed that in the orthodontic patients with a high caries risk, there were no statistically significant changes in the streptococcus mutans count between the three time points. The streptococcus mutans counts increased after the placement of orthodontic brackets/bands/wires for one month, then decreased slightly after one month’s use of probiotics. However, the differences were not statistically significant. Regarding the lactobacilli bacteria count, the main change occurred from T0 to T1. The use of probiotics for one month did not reduce the bacterial count of lactobacilli (T1 to T3). Overall, the effect of probiotics in orthodontic patients with high caries risk seemed to be less than in those with low caries risk.

### 4.4. Probiotics Selection and the Possible Mechanism of Bacterial Ecological Changes

It is a confounding phenomenon that Lactobacilli contained in probiotics are “good“ bacteria for intestinal microbial restoration, however, for the oral environment, lactobacilli are considered cariogenic and play a role in the progression of dental caries. However, not all lactobacilli species are cariogenic. According to the clinical review by Caufield et al., they listed and ranked the abundance of the lactobacillus species found in children and adults with dental caries. Therefore, in our study, we further used the VITEK^®^ MS (bioMérieux co., Marcy-l’Étoile, France) automated mass spectrometry microbial identification system to identify the species cultured from the light green agar (for lactobacilli) from the saliva of these orthodontic patients after one month of probiotics use. The data are illustrated in the supplementary file (Table S1). In fact, the bacterial species used in the probiotics were not identified in the saliva of the patients. 

The probiotic used in this study was Biofermin with the main components Lactobacteria 3 mg (Streptococcus faecalis) and Glycobacateria (bacillus natto) 2 mg. The choice was due to its availability. Patients were instructed to chew the tablets thoroughly before swallowing. If the coaggregation and competitive exclusion hypothesis shown in the literature review of Redi are true, the components of the probiotics used should be identified in the saliva of orthodontic patients after the use of probiotics. Through the microbial identification of this study, coaggregation and competitive exclusion may not be the direct mechanism for the inhibitory effect. However, Bacillus natto may play a role in biofilm formation. Kolodkin-Gal et al. found that Bacillus subtilis inhibited the formation of bacterial biofilms and triggered the scattering of mature biofilms by synthesizing D-tyrosine, D-leucine, D-methionine, and D-tryptophan [18]. The study by Iwamoto et al. stated that natto made from soybeans cultured with Bacillus subtilis natto inhibits the formation of biofilm [19]. The study of Martin et al. showed that Bacillus Natto was able to destroy the preformed biofilm [20], which might explain the mechanism by which the probiotics used in our study worked. However, future research is necessary to identify the mechanisms of probiotics in the oral microorganism.

### 4.5. Weaknesses and Limitations

The limitations of this study were as follows: (1) the use of bacteria count as outcome variable. However, in the clinical situation, the new occurrence of dental caries can be used as an outcome variable; (2) CRT^®^ kit (caries risk test) couldn’t give the exact colony count of bacteria or the accurate number of cariogenic bacteria when compared to the conventional culture-based assay; (3) the bacteria count was analyzed only one month after the use of probiotics. However, since participants in this study continued to use probiotics during orthodontic treatment procedure, in future research it would be useful to determine the long-term effect of probiotics in orthodontic patients and to use the occurrence of white spot lesions or new dental caries as an outcome variable, which would be more relevant to the clinical practice. The strength of this study was that patients were categorized into high and low risk groups, and thus the impact of probiotic use on these two groups could be further analyzed.

## 5. Conclusions

The placement of orthodontic brackets, bands, and wires in the oral cavities increased Streptococcus mutans and Lactobacilli counts one month after treatment. The use of probiotics (main component Lactobacteria 3 mg, Glycobacteria 2 mg/tab) decreased the bacteria counts of both cariogenic bacteria. However, the differences were not statistically significant. It was noteworthy that, even in patients with low caries risk, cariogenic bacteria count increased after one month of orthodontic treatment. Supplemental use of probiotic oral tablets during orthodontic treatment aimed at reducing cariogenic bacteria count in saliva did not achieve significant differences, regardless of whether patients were in the high or low caries risk group.

## Figures and Tables

**Figure 3:**
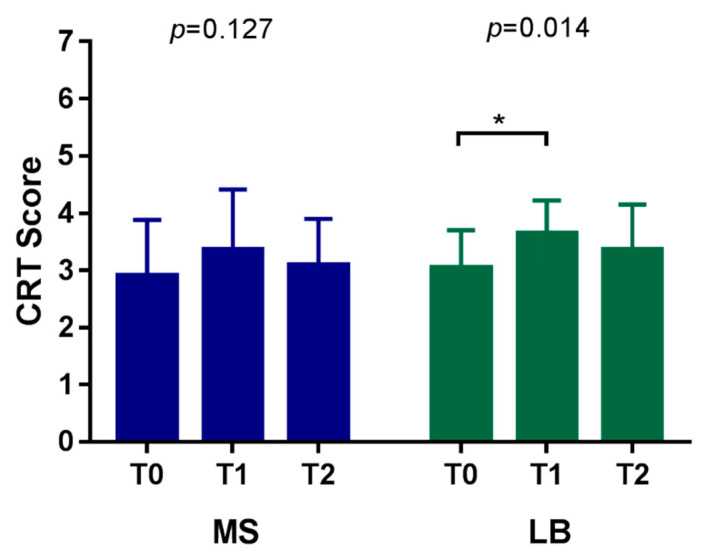
CRT score of the high caries risk group among three time points. Multiple comparison test. * *p* < 0.05.

**Table 1:** Changes in cariogenic bacterial count scores over time and multiple comparisons between three time points. (N = 33).

	T0		T1		T2		*p* Value ^a^	Dunn-Bonferroni Post Hoc ^b^		
	Mean	SD	Mean	SD	Mean	SD		T0 vs. T1	T0 vs. T2	T1 vs. T2
MS CRT score ^c^	2.45	1.06	3.21	1.11	3.03	0.92	0.001 **	0.011 *	1	0.109
LB CRT score ^c^	2.55	0.94	3.55	0.75	3.18	0.88	<0.001 **	0.001 **	0.372	0.109

^a^: Friedman test * *p* < 0.05, ** *p* < 0.01; ^b^: Post-hoc analysis (Dunn-Bonferroni) * *p* < 0.05, ** *p* < 0.01; ^c^: MS CRT score: Streptococcus mutans CRT^®^ bacteria kit evaluation chart with score modification; LB CRT score: lactobacilli species CRT^®^ bacteria kit evaluation chart with score modification in Figure 1.

**Table 2:** Comparison of high and low caries risk groups in gender, age, and bacteria counts at different time points.

	Low Caries Risk Group (*n* = 11)	High Caries Risk Group (*n* = 22)	*p* Value
Gender			0.711
M	5 (45.5%)	13 (59.1%)	
F	6 (54.5%)	9 (40.9%)	
Age	15.27 ± 3.23	15.68 ± 3.14	0.576
T0MS	1.55 ± 0.52	2.91 ± 0.97	<0.001 **
T0SB	1.55 ± 0.52	3.05 ± 0.65	<0.001 **
T1MS	2.91 ± 1.22	3.36 ± 1.05	0.232
T1SB	3.36 ± 1.03	3.64 ± 0.58	0.613
T2MS	2.91 ± 1.14	3.09 ± 0.81	0.716
T2SB	2.82 ± 0.98	3.36 ± 0.79	0.115

Mann–Whitney U test. * *p* < 0.05, ** *p* < 0.01. Continuous data are expressed as mean ± SD.

**Table 3:** CRT score of the low caries risk group among three time points.

Bacteria Count		CRT Score	*p* Value	Multiple Comparison		
				*p* Value		
MS count	T0	1.55 ± 0.52	0.008 *	T0–T1	T1–T2	T0–T2
	T1	2.91 ± 1.22		0.024 *	1	0.020 *
	T2	2.91 ± 1.14				
LB count	T0	1.55 ± 0.52	0.001 *	0.001 *	0.933	0.021 *
	T1	3.36 ± 1.03				
	T2	2.82 ± 0.98				

Kruskal–Wallis Test. * *p* < 0.05. Multiple comparison test. * *p* < 0.05, ** *p* < 0.01.

**Table 4:** CRT score of the high-caries-risk group among three time points.

Bacteria Count		CRT Score	*p* Value	Multiple Comparison		
				T0–T1	T1–T2	T0–T2
MS count	T0	2.91 ± 0.97	0.127			
	T1	3.36 ± 1.05				
	T2	3.09 ± 0.81				
LB count	T0	3.05 ± 0.65	0.014 *	0.011 *	0.256	0.717
	T1	3.64 ± 0.58				
	T2	3.36 ± 0.79				

Kruskal–Wallis Test. * *p* < 0.05, ** *p* < 0.01. Multiple comparison test. * *p* < 0.05.

## Data Availability

The data in this study are not publicly available but can be requested from the corresponding author.

## Supplementary Materials

The following supporting information can be downloaded at: https://www.mdpi.com/article/10.3390/nu14153196/s1, Table S1: Species identified from the CRT kit light green agar with saliva from the patients after one month of probiotics use.
