# Supplementary material for: The Effect of Probiotics Use on Salivary Cariogenic Bacteria in Orthodontic Patients with Various Caries Risk Status
Source: Nutrients. 2022 Aug 4;14(15):3196. doi: 10.3390/nu14153196 (PMC9370778; doi:10.3390/nu14153196)
Supplement: Supplementary file 1 [file nutrients-14-03196-s001.zip › nutrients-1805417-supplementary.pdf]

**Table S1. Species identified from the CRT kit light green agar with saliva from the patients after one month of probiotics use**
Lactobacillus fermentum
Lactobacillus pentosus/ plantrum/ paraplantrum
Enterobacter hormaechei
Klebseilla pneumonia
Lactobacillus brevis
Lactobacilli caseri//paracasei/rhamnosus
Staphylococcus haemolyticus
